# Healthcare system navigation difficulties among informal caregivers of older adults: a logistic regression analysis of social capital, caregiving support and utilization factors

**DOI:** 10.1186/s12913-024-11549-0

**Published:** 2024-10-01

**Authors:** Boah Kim, Andrew Wister, Barbara Mitchell, Lun Li, Laura Kadowaki

**Affiliations:** 1 Department of Gerontology, Simon Fraser University, 2800-515 Hastings Street, Vancouver, BC V6B 5K3 Canada; 2 Gerontology Research Centre, Department of Gerontology, Simon Fraser University, 2800-515 Hastings Street, Vancouver, BC V6B 5K3 Canada; 3 Department of Sociology & Anthropology, Simon Fraser University, 8888 University Drive, Burnaby, BC V5A 1S6 Canada; 4 School of Social Work, MacEwan University, 9-510A2, 10700 104 Ave NW, Edmonton, AB T5J 4S2 Canada

**Keywords:** System navigation, Informal caregivers, Older adults, Care coordination, Community and health system access

## Abstract

**Background:**

Informal caregivers of older adults play a vital role in improving the degree to which older adults access community and healthcare services in a seamless and timely manner. They are fulfilling important navigation and support roles for their older care recipients. However, there is still little knowledge of the most significant facilitators and barriers to effective and efficient system navigation among caregivers. This paper aims to fill these knowledge gaps through investigation of the key factors (i.e., social capital/cohesion, caregiving supports, and utilization factors) affecting navigation difficulties faced by informal caregivers of older adults.

**Methods:**

The Behavioural-Ecological Framework of Healthcare Access and Navigation (BEAN) model is used to frame the study. Using the General Social Survey on Caregiving and Care Receiving 2018, we analyzed 2,733 informal caregivers whose primary care recipients were aged 65 or older. Hierarchical logistic regression was conducted to identify the relationship between system navigation difficulties among informal caregivers and four sequentially ordered blocks of predictors: (1) sociodemographic (2), social capital/cohesion (3), caregiving supports, and (4) healthcare demand.

**Results:**

The fully adjusted model showed that the probability of reporting navigation difficulties was lower for caregivers with social capital/cohesion compared to those without social capital/cohesion. In comparison, the probability of reporting navigation difficulties was higher among caregivers with caregiving support and among caregivers whose care receivers use a higher amount of health service use. Several sociodemographic covariates were also identified.

**Conclusion:**

Our findings support certain aspects of the BEAN model. This study extends our understanding of potential facilitators and barriers that informal caregivers of older adults face while navigating complex community and health systems. There is a need to implement coordinated schemes and health policies especially for older adults with mental/neurological issues to address the challenges of their caregivers given the specific vulnerability identified in this study. The need for further research using different approaches to examine the disproportionate impact of COVID-19 on caregivers’ system navigation experience is crucial.

## Background

Healthcare system navigation (SN) can be defined as the process by which older adults and/or their health caregivers transition through the healthcare system in order to access and use services to maximize positive health outcomes [1]. Thus, navigation is a set of dynamic processes through which individuals respond to healthcare needs, pursue opportunities, and manage constraints [2]. As older adults experience an increase in the likelihood of chronic diseases, including physical/mental health problems and multimorbidity [3–7], they tend to require greater use of a variety of care providers while they are navigating community and healthcare systems to fulfill their complex health needs. Existing healthcare systems, however, are poorly suited to address older adults’ complex, wide-ranging and changing needs due to siloed system-level organizational structures, thereby posing significant challenges to older adult users and their caregivers [8, 9]. Specifically, the challenges include a lack of awareness of available services in care settings among health professionals and caregivers [10, 11] and an incomplete transfer of information among stakeholders due to miscommunications [12]. Thus, older adults and their caregivers often fall through the cracks due to a lack of information on care service options and fragmented medical information. This may prevent them from accessing various types of services in community and healthcare settings. Community care includes respite care, adult day services, and activity programs provided in home and communities, whereas healthcare services cover GPs/specialists service from hospitals, pharmacare, and ancillary services. Navigating and accessing necessary services in complex systems with such issues may present significant challenges in managing and coordinating care delivery to ensure optimal outcomes for older adults [13, 14]. Against this backdrop, informal caregivers, who have been recognized as the backbone of the healthcare system [15–17], often play a vital role in improving the degree to which older adults access community and healthcare services in a more seamless and timely manner. Informal caregivers are fulfilling important navigation and support roles for older care recipients given that they are providing unpaid adult care to about 35% of care receivers in Canada [16, 18–20]. This pattern is intensified by rapid population aging, whereby 19% of the population in 2021 was aged 65 and over, with an estimated 1 in 4 Canadians in this age category by 2041 [21, 22]. Yet, there is still little knowledge on the most significant facilitators and barriers to effective and efficient SN among caregivers, which is fundamental to reducing disparities in community and healthcare access for this focused group. This paper aims to fill these knowledge gaps through an investigation of the key factors affecting navigation difficulties faced by informal caregivers when accessing and navigating services for their older adult care-receivers.

**Fig. 1:**
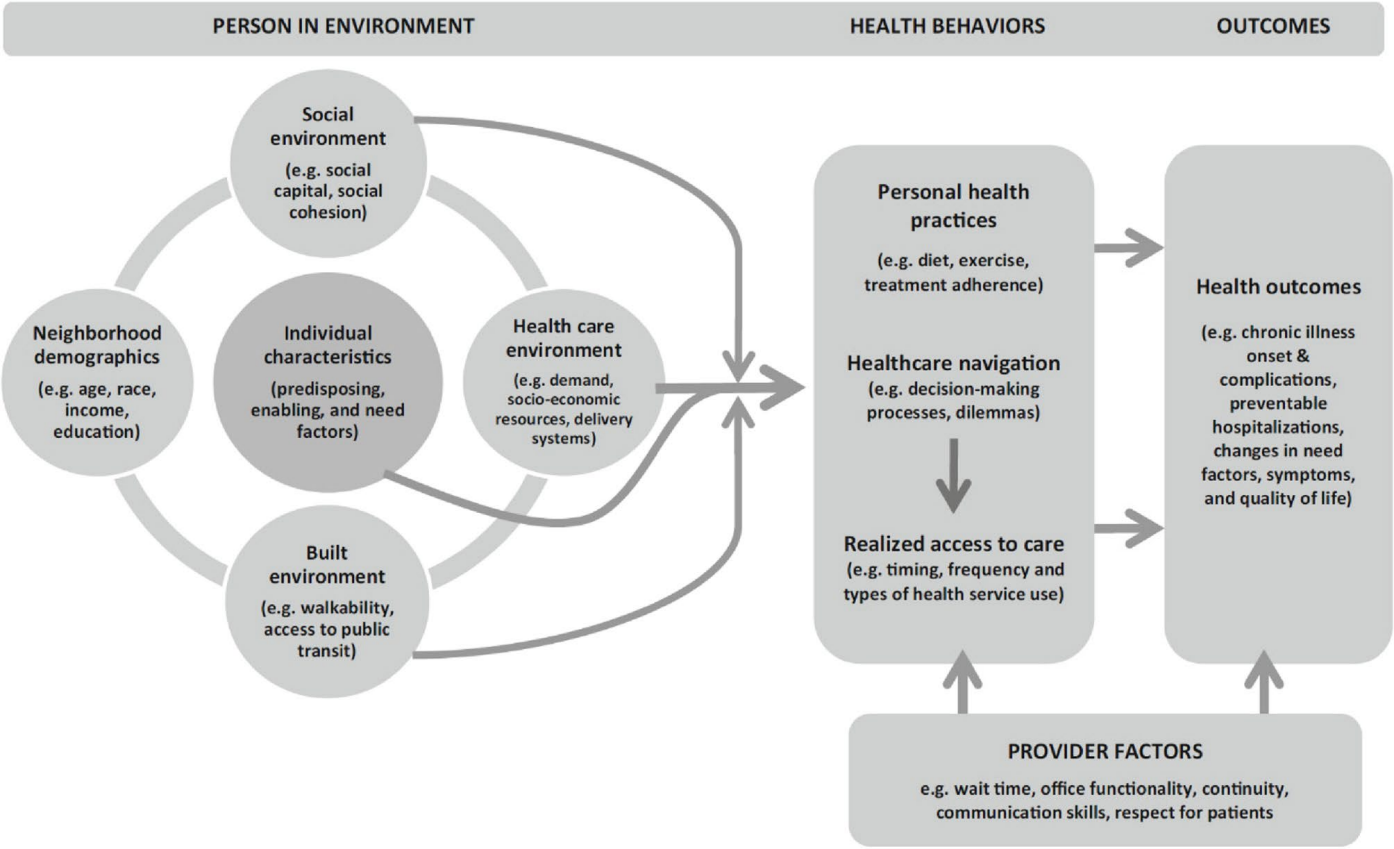
Behavioral-ecological framework of healthcare access and navigation. Source: Ryvicker (2018)

We frame this research using the Behavioural-Ecological Framework of Healthcare Access and Navigation (BEAN) developed by Ryvicker (2018) to understand and explore the factors that support and hinder SN [2]. The model integrates two models found in gerontology—namely, the Behavioural Model of Health Service Use [23] and the Ecological Model of Aging [24]. The model (Fig. 1) includes the basic structure of the Behavioural Model of Health Service Use while highlighting important environmental domains of influence, such as social and healthcare environment. Building on the Behavioural Model of Health Service Use, the BEAN posits that the complex interactions among an individual’s predisposing, enabling, and need characteristics influence a person’s health behaviour, which includes health practices (e.g., diet, exercise), SN processes and accessibility to care. Building on the ecological model of aging [24, 25], the BEAN focuses on three categories of environmental characteristics in which the individual is embedded: the social environment (e.g., social capital/cohesion), the healthcare environment (e.g., health service demand), and the built environment (e.g., walkability). This model helps to identify, organize and interpret the key factors affecting SN difficulties among informal caregivers of older adults. This paper proposes to investigate the association between navigation difficulties and the social capital of informal caregivers along with other factors (i.e., caregiving support and utilization factors). This aspect of SN specifies the significance of informal caregivers in the BEAN model. The model notes that informal caregivers should be involved in the navigation process stating that “informal caregiver support has been identified as important factors in navigation” (Ryvicker, 2018: page 228). Recognizing that informal caregivers provide instrumental (e.g., locating services, driving), informational (health knowledge and literacy), and emotional support in SN of older adults, it is critical to identify factors affecting related challenges among informal caregivers.

Studies have highlighted the advantage of social capital underlying caregiving access and navigation of the healthcare system [26, 27]. One case study found that the community-based organization supports socio-economically vulnerable people by providing various social capital resources (using a measure of mobilizing accessibility resources through informal relationships with friends and neighbours), which was found to be key in enabling navigation of healthcare during the COVID-19 pandemic [27]. Volunteers of the organization connected individuals to health services in various ways, relying on informal personal contacts within public services. This study demonstrated that linking social capital/cohesion and caregiving supports can foster more effective navigation practices. Another study [26] identified that system knowledge can be operationalized through social networks of trust and privilege (i.e., social capital). Researchers conducted in-depth, semi-structured interviews to explore the perspectives of participants pertaining to navigating the healthcare system and the knowledge that they used to make choices. They found that system knowledge requires social capital that can provide an advantage in utilizing the healthcare system. One of the participants of the study, for example, described how they sought information from their networks to ‘check the reputation’ of medical specialists. They noted that they were able to find suitable service providers effectively through their reliable social networks. The study illustrates that having trustworthy/reliable people (i.e., social capital) and/or caregiving support when having problems can facilitate the navigation of complex community and healthcare systems. Other studies have also noted that social support positively affects the SN [28–30], although many of these studies have focused on younger people or refugees of any age rather than older adults or caregivers.

Conversely, some research has found that the effects of social support on SN might not always be straightforward, and in fact, can enable or hinder SN in some instances [2]. For instance, a cohesive social support system can be an obstacle to effective SN in an environment in which distrust of health professionals can occur, due to past experiences and cultural differences [2, 31, 32]. Thus, the role of social capital and/or caregiving supports in SN is under-researched and findings remain equivocal, suggesting the need for further study.

Healthcare navigation for caregivers is also likely linked to the care demands of older care recipients, since their health context can affect the amount of healthcare needed. Many studies have shown that functional limitations are associated with health service utilization [33–37]. For instance, Rhee and colleagues (2020) found that multimorbidity (i.e., more than one concurrent chronic condition) was associated with higher healthcare among older adults. Specifically, they found that older adults with more severe functional limitations (disability) use a higher amount of health services to fulfill their complex health needs than those with less functional limitations, and concluded that this raises the risk for navigation challenges [34].

Other individual characteristics such as education level, health literacy, communication skills, and degree of self-efficacy have been identified as important factors in SN [1, 31, 32, 38–40]. A higher level of education is associated with enhanced health literacy and confidence in communicating with health professionals [39, 40], which may positively facilitate one’s SN process. In addition, psycho-social factors that have been associated with higher use of services (such as health-related self-efficacy and depression) can either improve SN or hinder effective SN, respectively, as has been shown for self-management of chronic conditions [38].

In summary, while several studies have been conducted to examine the potential factors of difficulties in SN among older adults, there is a dearth of empirical studies that fully explore facilitators and/or barriers to SN among informal caregivers. This study will specifically identify the social capital, caregiving support and utilization factors that can facilitate or act as a barrier to the effective use of community and healthcare services among informal caregivers of older adults. It examines a specific aspect of the BEAN model focusing on the social environment and individual characteristics among informal caregivers as potential facilitators and/or barriers to SN.

The hypotheses of this study are as follows:

1. Higher levels of social capital/cohesion among informal caregivers will be associated with less difficulties in SN.
2. Higher levels of caregiving support among informal caregivers will be associated with less difficulties in SN.
3. Greater amounts of health service utilization among older care receivers will be associated with more difficulties in SN among informal caregivers.

## Methods

### Design and sample

This study employs the General Social Survey (GSS) on Caregiving and Care Receiving 2018 (Cycle 32) (hereafter: GSS 32). This survey collected information between April 3rd to December 28th, 2018 on Canadians who received help or care because of a long-term health condition, a disability or problems related to aging, and those who provide help or care to family members or friends with these types of conditions. The target population includes all persons 15 years of age and older in Canada, excluding (1) residents of the Yukon, Northwest Territories, and Nunavut; (2) full-time residents of institutions. The final sample size for GSS 32 was 20,258.

The definition of informal caregivers for this study entails: (1) a person who has helped or cared for someone with a long-term health condition or a physical/mental disability or problems related to aging, (2) a person who provided care at least 1 h per week, (3) whose primary care receiver had received help from professionals, that is paid workers or organizations, and (4) whose primary care receiver are aged 65 or older. Based on the criteria, the final sample size of this study is 2,733.

### Measures

#### Outcome variable

The primary outcome variable used to measure community and healthcare navigation problems is a binary response (yes, no) to a question asking: “what specifically did you find stressful about caregiving: Finding services for your care receiver(s).” The majority of the sample (81%) reported having no difficulties while 19% reported in the affirmative (see Table 1).

**Table 1:** Characteristics of informal caregivers and their care receivers, weighted

**Dependent Variable**	**Percentage (%)**			
Navigation difficulties				
No	81.01			
Yes	18.99			
**Independent variables**	**Percentage (%)**			
	**Entire Study population**	**Having Navigation difficulties**	**Not Having Navigation difficulties**	**Chi-square** **(df)**
*Informal Caregiver Variables* Sex Female Male	60.37 39.63	68.59 31.41	58.45 41.55	18.10 (1) ***
Age Under 45 years old 45 to 64 years old 65 years and older	17.09 51.48 31.43	13.68 59.92 26.40	17.89 49.50 32.61	18.38 (2) ***
Education Below high school High school or equivalent College diploma/certificate University degrees or above	7.74 32.20 28.24 31.82	2.94 30.00 30.98 36.08	8.88 32.73 27.58 30.81	25.01 (3) ***
Personal annual income Less than $40,000 $40,000 to $80,000 More than $80,000	43.51 34.39 22.10	43.16 34.30 22.54	43.59 34.42 22.00	0.08 (2)
Marital status Not married Married or common law	36.22 63.78	34.30 65.70	36.68 63.32	1.03 (1)
Employment No Yes	50.05 49.95	53.37 46.63	49.14 50.86	3.01 (1)
Emotional health and well-being Unhappy Happy	8.71 91.29	10.98 89.02	8.18 91.82	4.17 (1) *
Self-rated health Good/Excellent Poor/Fair	84.64 15.36	78.36 21.64	86.11 13.89	19.20 (1) ***
Self-rated mental health Good/Excellent Poor/Fair	86.68 13.32	80.86 19.14	88.05 11.95	18.58 (1) ***
Self-rated stress Not stressful Stressful	31.46 68.54	16.73 83.27	34.94 65.06	63.94 (1) ***
Long-term health condition/disability No Yes	78.18 21.82	75.44 24.56	78.82 21.18	2.77 (1)
Visible minority No Yes	92.67 7.33	92.20 7.80	92.78 7.22	0.21 (1)
Primary caregiver No Yes	58.43 41.57	43.55 56.45	61.92 38.08	58.47 (1) ***
Relationship with care receiver Spouse Immediate family Family in-law Others	14.34 57.85 12.73 15.44	15.41 64.16 14.07 6.36	14.09 55.92 12.42 17.57	40.60 (3) ***
Social capital/cohesion No More or less Yes	13.56 26.64 59.80	20.51 31.84 47.66	11.91 25.41 62.68	44.57 (2) ***
Caregiving supports No Yes	72.83 27.17	63.94 36.06	74.93 25.07	24.78 (1) ***
Healthcare demand (formal health service use) Less than 1 h 1 h to less than 3 h 3 h to less than 5 h 5 h to less than 10 h 10 h or more Not stated	21.81 25.21 10.65 9.29 15.66 17.38	17.53 25.43 10.98 13.29 23.89 8.86	22.81 25.16 10.57 8.36 13.73 19.38	70.94 (5) ***
*Care Receiver Variables* Sex Female Male	64.50 35.50	66.80 33.20	63.96 36.04	1.47 (1)
Age 65 to 74 years 75 to 84 years 85 years and older	24.19 36.35 39.46	20.59 30.88 48.53	24.96 37.52 37.52	17.05 (2) ***
Employment status receivers No Yes	95.15 4.85	95.38 4.62	95.09 4.91	0.07 (1)
Living arrangement Living in the same household Not living together	22.70 77.30	26.11 73.89	21.91 78.09	4.23 (1) *
Main Health Condition Chronic issues and/or disability Mental and neurological issues Aging and frailty Others	42.96 14.52 27.02 15.51	44.12 19.46 22.16 14.26	42.69 13.35 28.16 15.80	17.24 (3) ***

#### Predictor variables

**Fig. 2:**
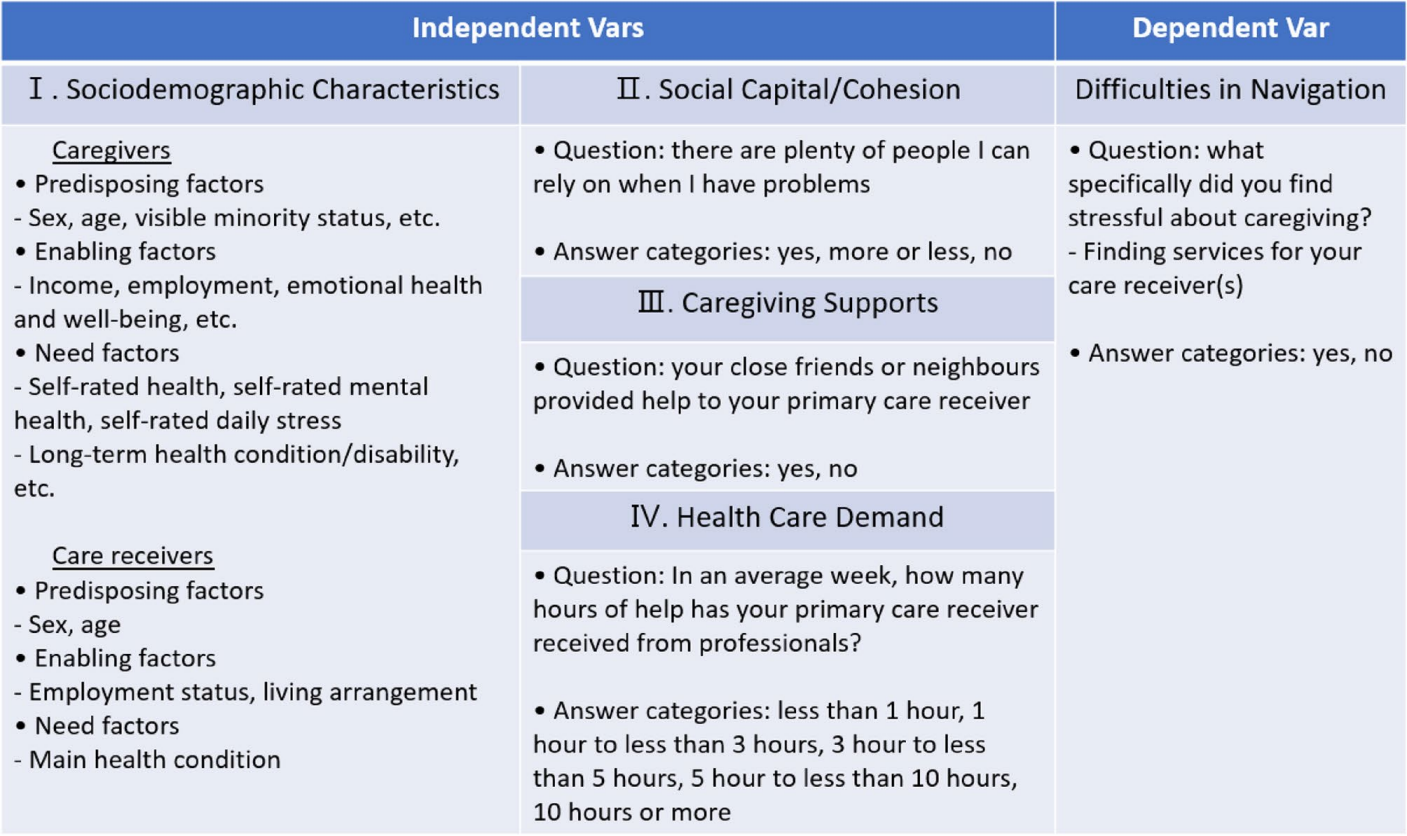
Variables

The independent variables used for the analysis encompass four domains: (1) sociodemographic variables (caregivers/care receivers), (2) social capital/cohesion variables, and (3) caregiving supports variables, and (4) healthcare demand variables (see Fig. 2).

#### Sociodemographic variables-caregivers

Predisposing factors (e.g., sex, age, visible minority status), enabling factors (e.g., marital status, income, employment, emotional health and well-being), and need factors (e.g., self-rated health, self-rated mental health, long-term health condition/disability) were used as socio-demographic individual characteristics of caregivers. Sex is a dichotomous variable coded with males as the reference category. Age is a categorical variable grouping informal caregivers into three groups (under 45/45 to 64/ over 65). The education level variable was measured with the following dummy coded categories: “below high school,” (reference) “high school or equivalent,” “college diploma/certificate,” and “university degrees or above.” Personal annual income was also dummy coded using the available categories in the dataset as possible responses: “less than $40,000,” (reference) “$40,000 to $80,000,” “$80,000 and over.” Marital status and employment status were dichotomized as “not married,” (reference) “married or common-law” in the case of the former variable, “no” (reference) and “yes,” for the latter variable. Emotional health and well-being was also dichotomized as “unhappy,” (reference) “happy”. Self-rated health and self-rated mental health were recoded from the original scale to “poor/fair” (reference) and “good/excellent, while self-rated stress was recoded to “not stressful” (reference) and “stressful.” Variables measuring their long-term health condition, visible minority status, and whether being a primary caregiver or not were used as it is in the original scale. Relationship with the (primary) care receiver was recoded from the original scale to “spouse,” “immediate family,” “family-in-law,” and the reference category “others.”

#### Sociodemographic variables-primary care receivers

Sex at birth was a dichotomous variable measured by “female” and “male,” with males being used as the reference category. Age is measured using three groups (65 to 74/75 to 84/ over 85), which were subsequently re-coded with the lowest age as the reference category. Employment status was dichotomized as “no” (reference) and “yes.” Living arrangement was dichotomous variable with “not living together” (reference) and “living in the same household.” Main health condition was measured with the following dummy coded categories: “aging and frailty,” (reference) “chronic issues and/or disability,” “mental and neurological issues,” and “others.”

#### Social capital/cohesion variable

For social capital/cohesion, the original scale “there are plenty of people I can rely on when I have problems” was used, of which the measure was “no,” (reference) “more or less,” and “yes.”

#### Caregiving supports variable

In terms of caregiving supports, the answer category “your close friends or neighbours provided him/her with help” was utilized to indicate the caregiving supports given to informal caregivers while navigating the systems for their older care receivers, which was asked by the original question “to accommodate your caregiving duties, has any of the following support been provided to you?”. It was measured as a dichotomous variable with “no” (reference) and “yes.”

#### Healthcare demand variable

The number of hours of help from health professionals that the primary care receiver received was used as an indicator, which was measured with the following answer categories: “less than 1 hour,” (reference) “1 hour to less than 3 hours,” “3 hour to less than 5 hours,” “5 hour to less than 10 hours,” and “10 hours or more.”

### Data analysis

Descriptive analyses were performed to summarize the characteristics of older adults and their informal caregivers. Additionally, hierarchical logistic regression was conducted to identify the relationship between SN difficulties among informal caregivers (outcome variable) and four sequentially ordered blocks of predictors: [1] sociodemographic [2], social capital/cohesion [3], caregiving supports, and [4] healthcare demand [41]. Hierarchical logistic regression is appropriate for modelling binary dependent variables such as ours [42]. Model 1 consisted of sociodemographic variables. Model 2 included the social capital/cohesion variable in addition to all variables in Model 1. Model 3 comprised all variables in Model 2 adding the caregiving supports variable. The final Model 4 constituted all variables in previous models in addition to healthcare demand variable. Each chi-square value of the model was presented to show the change across the four models. All analyses were conducted with STATA version 18.0.

## Results

### Descriptive statistics

#### Characteristics of informal caregivers and their older care receivers

Table 1 shows the characteristics of informal caregivers and their older care receivers. For the caregivers, 60.37% were female. Close to half (51.48%) were 45 to 64 years old. About 28% had a college diploma/certificate and over 40% had an income of less than $40,000. In addition, 36.22% were not married and almost half of respondents (49.95%) were employed. Most (91.29%) reported that they were happy with their emotional health. About 85% perceived their health or mental health to be good/excellent (84.64% and 86.68%, respectively), whereas almost 69% felt stressed. Also, 21.82% had a long-term health condition/disability. A small percentage (7.33%) were a visible minority. A significant proportion (41.57%) were primary caregivers and over half of the respondents (57.85%) were immediate family members of their older care receivers. We found that 13.56% had social capital/cohesion and 27.17% had caregiving supports while navigating the community and healthcare system. About a quarter of respondents’ care receivers used 1 h to less than 3 h of formal health service use. In the case of older care receivers, 64.50% were female and almost 40% were 85 years and older. The majority of care receivers were not employed (95.15%). Also, 22.70% lived with their caregivers. Over 42% had chronic issues and/or disability.

#### Navigation difficulties among Informal caregivers of older adults

Table 1 also presents the proportion of informal caregivers who had navigation difficulties. Almost 19% of caregivers (18.99%) faced challenges while navigating the community and healthcare system for their older care receivers, while about 81% (81.01%) did not have navigation difficulties.

### Factors associated with navigation difficulties among informal caregivers

Table 2 shows the results on factors associated with navigation difficulties among informal caregivers of older adults. All four models were significant (*p* < .001) and the chi-square of each model was presented (Model 1: 252.21, Model 2: 266.55, Model 3: 283.62, and Model 4: 320.64). Only results in the final model (Model 4) are presented for each section below, although all hierarchical models are displayed in the tables. SN difficulties (yes/no) was significantly associated with 11 variables. Only statistically significant associations are reported below.

**Table 2:** Hierarchical logistic regression of navigation difficulties among informal caregivers of older adults (*n* = 2,733)

	Model 1	Model 2	Model 3	Model 4
	Odds Ratio (95% C.I.)	Odds Ratio (95% C.I.)	Odds Ratio (95% C.I.)	Odds Ratio (95% C.I.)
***1) Sociodemographic model***				
***Caregivers***				
**Sex** (male—ref) Female	1.41** [1.09 to 1.82]	1.42** [1.10 to 1.85]	1.44** [1.10 to 1.88]	1.42* [1.08 to 1.87]
**Age** (Under 45 years—ref) 45 to 64 years 65 years and older	1.42 [0.96 to 2.09] 1.44 [0.87 to 2.34]	1.35 [0.91 to 2.01] 1.39 [0.85 to 2.27]	1.38 [0.92 to 2.05] 1.54 [0.94 to 2.54]	1.39 [0.92 to 2.09] 1.54 [0.93 to 2.55]
**Education level** (below high school—ref)				
high school or equivalent	4.39*** [2.03 to 9.51]	4.11*** [1.89 to 8.96]	4.04*** [1.85 to 8.85]	3.93*** [1.79 to 8.65]
college diploma/certificate	5.25*** [2.42 to 11.41]	4.89*** [2.24 to 10.68]	4.91*** [2.24 to 10.76]	4.91*** [2.23 to 10.83]
university degrees or above	7.32*** [3.35 to 16.02]	6.67*** [3.35 to 14.67]	6.31*** [2.86 to 13.93]	5.96*** [2.68 to 13.22]
**Personal annual income** (less than $40,000—ref)				
$40,000 to $80,000	0.84 [0.63 to 1.11]	0.85 [0.64 to 1.13]	0.87 [0.65 to 1.17]	0.91 [0.68 to 1.23]
$80,000 and over	0.78 [0.56 to 1.10]	0.82 [0.58 to 1.15]	0.89 [0.62 to 1.26]	0.95 [0.67 to 1.36]
**Marital status** (not married—ref) Married or common-law	1.10 [0.83 to 1.46]	1.14 [0.86 to 1.51]	1.20 [0.90 to 1.61]	1.20 [0.89 to 1.61]
**Employment status** (No—ref)				
Yes	1.17 [0.88 to 1.55]	1.18 [0.89 to 1.56]	1.19 [0.89 to 1.59]	1.17 [0.87 to 1.57]
**Emotional health and well-being** (unhappy—ref) Happy	1.06 [0.67 to 1.67]	1.09 [0.68 to 1.76]	1.06 [0.65 to 1.74]	1.10 [0.66 to 1.82]
**Self-rated health** (poor/fair—ref) Good/Excellent	0.62** [0.44 to 0.88]	0.65* [0.46 to 0.93]	0.66* [0.46 to 0.94]	0.66* [0.46 to 0.95]
**Self-rated mental health** (poor/fair—ref) Good/Excellent	0.79 [0.54 to 1.16]	0.87 [0.59 to 1.29]	0.90 [0.60 to 1.36]	0.90 [0.60 to 1.36]
**Self-rated stress** (not stressful—ref) Stressful	2.51*** [1.83 to 3.44]	2.35*** [1.71 to 3.23]	2.26*** [1.63 to 3.14]	2.24*** [1.61 to 3.13]
**Long-term health condition** (no—ref) Yes	1.05 [0.79 to 1.40]	1.02 [0.76 to 1.35]	1.04 [0.78 to 1.40]	0.91 [0.68 to 1.23]
**Visible minority status** (visible minority—ref) Not a visible minority	0.96 [0.62 to 1.48]	1.04 [0.67 to 1.61]	1.13 [0.71 to 1.79]	1.09 [0.81 to 1.47]
**Primary caregiver** (no—ref) Yes	2.40*** [1.82 to 3.16]	2.39*** [1.81 to 3.15]	2.34*** [1.77 to 3.11]	2.37*** [1.77 to 3.16]
**Relationship with the Care receiver** (others—ref) Spouse Immediate family Family in-law	1.53 [0.75 to 3.10] 2.49*** [1.54 to 4.02] 3.27*** [1.86 to 5.75]	1.54 [0.75 to 3.15] 2.53*** [1.55 to 4.12] 3.33*** [1.87 to 5.92]	1.46 [0.70 to 3.05] 2.69*** [1.63 to 4.45] 3.36*** [1.86 to 6.08]	1.44 [0.69 to 3.04] 2.61*** [1.57 to 4.35] 3.18*** [1.73 to 5.82]
***Primary care receivers***				
**Sex** (male—ref) Female	1.15 [0.88 to 1.50]	1.16 [0.88 to 1.51]	1.09 [0.83 to 1.43]	1.73 [0.81 to 1.41]
**Age of Care receivers** (65 to 74 years—ref) 75 to 84 years 85 years and older	0.94 [0.66 to 1.33] 1.54** [1.21 to 2.47]	0.94 [0.66 to 1.39] 1.74** [1.22 to 2.50]	0.95 [0.66 to 1.36] 1.87*** [1.29 to 2.72]	0.92 [0.64 to 1.34] 1.67** [1.14 to 2.44]
**Employment status of Care receivers** (no—ref) Yes	0.99 [0.53 to 1.83]	0.98 [0.53 to 1.82]	0.98 [0.52 to 1.84]	1.08 [0.58 to 2.03]
**Living arrangement of Care receivers** (not living together—ref) Living in the same household	1.07 [0.72 to 1.59]	1.04 [0.70 to 1.56]	1.03 [0.68 to 1.56]	1.05 [0.69 to 1.60]
**Main health condition** (aging and frailty—ref) Chronic issues and/or disability Mental and neurological issues Others	1.61* [1.01 to 2.59] 2.21*** [1.24 to 3.93] 1.70 [0.94 to 3.11]	1.55 [0.95 to 2.51] 2.30*** [1.30 to 4.05] 1.67 [0.92 to 3.03]	1.54 [0.93 to 2.56] 2.41*** [1.33 to 4.34] 1.76 [0.97 to 3.21]	1.56 [0.92 to 2.65] 2.09* [1.11 to 3.95] 1.82 [0.99 to 3.35]
***2) Social capital/cohesion model***				
**Having reliable people when having problems** (no—ref)				
More or less Yes		0.82*** [0.58 to 0.96] 0.55*** [0.39 to 0.78]	0.76*** [0.54 to 0.97] 0.47*** [0.33 to 0.67]	0.78*** [0.54 to 0.95] 0.45*** [0.32 to 0.65]
***3) Caregiving supports model***				
**Close friends/neighbours provide help** (no—ref)				
Yes			1.92*** [1.46 to 2.53]	1.90*** [1.44 to 2.50]
***4) Healthcare demand model***				
**Formal health service use** (less than 1 h—ref)				
1 h to less than 3 h 3 h to less than 5 h 5 h to less than 10 h 10 h or more Not stated				1.31 [0.92 to 1.89] 1.40 [0.87 to 2.25] 2.23*** [1.42 to 3.50] 2.86*** [1.93 to 4.24] 1.03 [0.63 to 1.68]
**Model Chi-Square (df)**	252.21*** (28)	266.55*** (30)	283.62*** (31)	320.64*** (36)

Model 1 = Sociodemographic variables; Model 2 = All variables in Model 1 plus social capital/cohesion variable; Model 3 = All variables in Model 2 plus caregiving supports variable; Model 4 = All variables in Model 3 plus healthcare demand var

*Ref* reference category, *CI* confidence interval

*p < .05, **p < .01, ***p < .001

#### Socio-demographic variables

The likelihood of reporting navigation difficulties (compared to not reporting) was higher for female caregivers than for male caregivers (OR = 1.42, *p* < .05, CI 1.08–1.87). The probability of having navigation problems was also associated with higher education levels for the following contrasts: “high school or equivalent,” (OR = 3.93, *p* < .001, CI 1.79–8.65) “college diploma/certificate,” (OR = 4.91, *p* < .001, CI 2.23–10.83) and “university degrees or above,” (OR = 5.96, *p* < .001, CI 2.68–13.22) compared to “below high school.” Better self-rated health was associated with a lower likelihood of having navigation difficulties: “good/excellent,” (OR = 0.66, *p* < .05, CI 0.46-0.95) compared to “poor/fair.” A higher likelihood of reporting navigation problems was found for reporting feeling “stressful,” (OR = 2.24, *p* < .001, CI 1.61–3.13) compared to “not stressful.”; and for being a primary caregiver: “yes” (OR = 2.37, *p* < .001, CI 1.77–3.16) compared to “no.” For the relationship with the primary care receiver, the higher likelihood of having navigation difficulties was reported for being “immediate family,” (OR = 2.61, *p* < .001, CI 1.57–4.35) “family-in-law” (OR = 3.18, *p* < .001, CI 1.73–5.82) compared to “others”.

For the care receivers-related variables, both age and main health condition showed significant positive relationships with navigation difficulties among caregivers. The likelihood of navigation problems was higher among informal caregivers whose primary care receivers were “85 years and older” compared to “65 to 74 years” (OR = 1.67, *p* < .01, CI 1.14–2.44). Also, the probability of reporting higher navigation difficulties was found for reporting having “mental and neurological issues” compared to “aging and frailty” (OR = 2.09, *p* < .05, CI 1.11–3.95).

#### Social capital/cohesion variables

The likelihood of navigation problems was lower among informal caregivers reporting more social capital/cohesion compared to those without social capital/cohesion. Specifically, the probability of reporting navigation difficulties was lower for caregivers who have comparatively higher level of social capital/cohesion (those who answered “more or less” or “yes”) (OR = 0.78, *p* < .001, CI 0.54-0.95; OR = 0.45, *p* < .001, CI 0.32-0.65, respectively) compared to those answering “no”.

#### Caregiving supports variables

The probability of reporting navigation difficulties was higher among caregivers with caregiving support compared to caregivers without caregiving support (OR = 1.90, *p* < .001, CI 1.44–2.50).

#### Healthcare demand variables

Healthcare demand was significantly associated with difficulties in SN. That is, the likelihood of reporting navigation difficulties was higher for caregivers whose care receivers use a comparatively higher amount of formal health service use: “5 hours to less than 10 hours,” (OR = 2.23, *p* < .001, CI 1.42–3.50) “10 hours or more” (OR = 2.86, *p* < .001, CI 1.93–4.24) compared to “less than 1 hour.”

## Discussion

The importance of community and healthcare system navigation (SN) among informal caregivers is vital to meeting the care needs of a growing older population, which is in its most rapid phase primarily due to the aging of the baby boomers and rising life expectancy [43, 44]. This paper extends our understanding of potential facilitators and barriers that informal caregivers of older adults face while navigating complex community and health systems. We specifically test several hypotheses pertaining to the importance of social capital/cohesion, caregiving supports, and healthcare demand, as well as examine several socio-demographic covariates.

In support of Hypothesis 1, our study revealed that informal caregivers with higher levels of social capital/cohesion reduces the likelihood of navigation difficulties, adjusting for all covariates. This finding is indicative of the potential benefits of social capital, such as providing reliable information and other forms of social support that may mitigate the problems associated with negotiating potentially challenging community and health systems. Indeed, researchers have noted that navigation challenges and caregiver burden can be compounded by vague or conflicting information on how to access public resources [9, 45]. Caregivers with stronger cohesion have also been found to be more likely to request timely, user-friendly guidance and information [8, 46, 47].

However, we also found that caregivers who received more help from their close friends/neighbours were more likely to face difficulties in SN, contrary to Hypothesis 2. This finding is consistent with the literature on this topic that has shown mixed results or even a possible “downside” of community/neighbourhood resources [2]. For example, a previous study found that higher levels of community social trust were associated with better self-rated health [48]; yet, this effect differed greatly depending on a person’s level of social trust, an indicator of social capital [2, 48]. For this study, it is possible to assume that reverse causation occurred. That is, informal caregivers dealing with navigation challenges might seek more support from their close friends/neighbours. Similar findings have been reported in other research examining the association between caregiving demands and community peer support use. Researchers of the study found that those with greater caregiving demands (measured by caregiving burden, time spent caregiving, total number of activities with which caregiver assists care receivers) were more likely to need peer support [49]. Our study uses the BEAN model and prior research in terms of our interpretation. Also, given that a substantial amount of literature stresses the importance of the roles of friends and neighbours in supporting older adults [49–54], it is assumed that the current variable to measure caregiving support is appropriate to verify hypothesis 2. Additionally, we contend that a significant reliance on informal social networks rather than on professional sources may disturb caregivers in obtaining proactive and timely information which is one of their significant information needs during the help-seeking process [55–57]. This is particularly relevant in our study, since the primary care receiver had to have received professional help. Yet, other sources and/or forms of social support not included in this study may actually reduce navigation problems.

In support of Hypothesis 3, our study found that higher healthcare demand of care receivers increased the navigation difficulties of their informal caregivers. Older care receivers who are using higher amounts of health services may have more complex social and health needs to be fulfilled. To address their complex needs, their informal caregivers often need to contact numerous community and health institutions and various professional care providers, which may cause significantly more challenges in the help-seeking process. While this finding is intuitive, it nonetheless emphasizes the importance of focusing on high-demand older adults with respect to healthcare navigation and integration.

Several sociodemographic indicators were also consistently associated with difficulties in SN. It is well established that female caregivers tend to be the most affected by caregiving [58–62]. Previous studies demonstrate that, particularly for women, entering a caregiving role reduces labour force participation [63, 64] as well as increases the probability of being retired [65]. Another recent study found a statistically significant positive association between transitioning into a caregiving role and increased network size among male caregivers, while female caregivers reported the opposite effect [62]. This may suggest that female caregivers are more likely to face greater challenges in SN, in part, due to the greater caregiving load and types of care, while having a smaller social network compared to male caregivers [66–68].

The positive relationship between caregivers’ education level and navigation difficulties differs from previous research in which education is a facilitator for more effective system use [39, 40]. Although speculative, the opposite association found in our study may be indicative of a higher level of health literacy and understanding of community and healthcare systems, which may raise expectations and increase perceptions of utilization problems, perhaps heightened due to higher rates of users. However, the role of education in SN should be investigated further. For caregivers with worse self-rated stress, and who are a primary caregiver may have more difficulties in SN due to their relatively vulnerable psychosocial and caregiving outcomes, while better self-rated health decreased navigation challenges among caregivers [69–71].

It was notable that the relationship with the care receiver had a significant association with navigation difficulties of caregivers. It is well established that taking care of family members causes a significant burden to caregivers compared to those who are taking care of others such as friends, neighbours or co-workers [51, 72–75]. Many studies have also demonstrated that caregivers who provide care to a chronically ill family member are at risk for caregiver burden and declining physical and mental health, factors that may constrain effective system use [73, 74, 76–80].

Our study also found that informal caregivers are likely to have higher navigation difficulties for taking care of the oldest old (85 years and older) compared to those providing care for the young-old (65 to 74 years). A significant number of studies have shown that older age is associated with a higher prevalence of multimorbidity, which can be a barrier to a negotiating community and health systems [81–84]. Accordingly, it is assumed that navigation difficulties among informal caregivers whose primary care receivers are the oldest-old may be higher, given that caregivers need to contact more community and healthcare institutions and stakeholders to address the complex needs of their care receivers.

It was also observed that informal caregivers whose care receivers have mental and neurological issues have greater difficulties than those whose care receivers have aging and frailty issues. Previous literature has found that older adults, and especially those living with dementia, can experience barriers in accessing and navigating fragmented service systems [10, 85–87]. In the case of older adults experiencing mental or neurological disease, their caregivers may misinterpret symptoms and declines in function as normal aging or grief [88]. In addition, Dawson and colleagues (2017) noted that delays in services were often experienced by caregivers in the face of mental health problems of their care receivers, since specialist services take more time. As such, individual-level problems stemming from the characteristics of mental/neurological disease significantly affect the SN. Furthermore, system-level factors such as continuity of care, wait time, and communication skills across the sectors are also critical to navigation experiences among informal caregivers and realized access to care [88].

Given the significance of the social capital and utilization factors, as well as several key covariates as predictors of navigation difficulties among informal caregivers, our findings support certain aspects of the BEAN model by Ryvicker (2018)—used to frame this study. That is, social capital/cohesion and health service use are identified as factors affecting healthcare navigation. Also, our study confirmed that some individual characteristics such as sex, education level, and self-rated stress affect the way in which caregivers navigate healthcare systems along with their care receivers’ characteristics (i.e., age, main health condition), which is aligned with the assumptions of the BEAN model. Indeed, the BEAN model is valuable for further understanding the effects of social capital/cohesion, healthcare demand, and several socio-demographic factors on accessing and navigating the community and healthcare system.

### Implications

In order to address the challenges and to fill the care gaps on SN among informal caregivers, there is a need to implement coordinated schemes and health policies for older adults and their caregivers, enabling user-friendly integrated care in community and healthcare systems. Potential strategies can focus on comprehensive personal and environmental assessment, care coordination matched to the level of client needs, and referral to additional service providers, etc. [89–91], thereby improving the navigation experience of informal caregivers as well. In doing so, there is also a need to pay more attention to older adults with mental/neurological issues to address the challenges of their caregivers given the specific vulnerability during the help-seeking process. Additionally, the COVID-19 pandemic has significantly affected not only the lives of older adults but also those of informal caregivers by increasing their level of stress, anxiety, and depression in care provision due to decreased support from both formal and informal sources [85, 92, 93]. Such adversity may negatively affect the experience of navigating the complex community and health systems among informal caregivers. Future research needs to further investigate these disproportionate impacts on caregivers. For example, qualitative studies can more deeply explore the SN challenges and/or experiences among informal caregivers during and after the COVID-19 pandemic.

#### Limitations

This study has several limitations. First, the cross-sectional design of the GSS 32 does not provide information on causal relationships. Thus, it may measure the existence and associations of relationships between independent variables and outcome variables that are present in the data collection environment. Still, this study fills the knowledge gap between the previous studies by focusing on navigation difficulties among informal caregivers, albeit in a cross-sectional design. Second, while the GSS 32 contains a wide variety of measures on caregiving and care receiving, the measure available for the outcome variable of the current study—navigation difficulties of informal caregivers—needs further development and specification. Third, not all of the components of the BEAN model were utilized. However, this study still contributes to aspects of the BEAN model by focusing on the social environment and individual characteristics of caregivers as potential facilitators/barriers to SN. Understanding the key predictors of navigation difficulties among informal caregivers can also be enhanced by including additional indicators of environmental areas (e.g., built environment), given our conceptual model [2]. Finally, SN problems are affected by the type and organization of health systems, as well as insurance, thereby necessitating research to be extended to other countries.

## Conclusions

Informal caregivers are fulfilling a pivotal navigator role for their older care receivers, thereby filling care gaps within often fragmented and complex community and healthcare systems. Our findings highlight that there are several key factors that are associated with navigation difficulties among caregivers in both social and healthcare environments. In particular, our study clearly demonstrates that coordinated initiatives and health policy should be developed to fully support informal caregivers and their older care receivers, especially those with greater and more complex health needs, to foster improved navigation experiences, such as seamless integrated care. This study serves as a stepping stone for research investigating the understanding and development of integrated care and comprehensive health policy strategies to reduce the navigation challenges among informal caregivers and older care receivers.

## Data Availability

All data generated or analysed during this study are included in this published article.

## Abbreviations

SN: System Navigation
BEAN model: the Behavioural-Ecological Framework of Healthcare Access and Navigation model
GSS: General Social Survey
